# Membership-Degree Preserving Discriminant Analysis with Applications to Face Recognition

**DOI:** 10.1155/2013/275317

**Published:** 2013-10-07

**Authors:** Zhangjing Yang, Chuancai Liu, Pu Huang, Jianjun Qian

**Affiliations:** School of Computer Science and Engineering, Nanjing University of Science and Technology, Nanjing 210094, China

## Abstract

In pattern recognition, feature extraction techniques have been widely employed to reduce the dimensionality of high-dimensional data. In this paper, we propose a novel feature extraction algorithm called membership-degree preserving discriminant analysis (MPDA) based on the fisher criterion and fuzzy set theory for face recognition. In the proposed algorithm, the membership degree of each sample to particular classes is firstly calculated by the fuzzy *k*-nearest neighbor (FKNN) algorithm to characterize the similarity between each sample and class centers, and then the membership degree is incorporated into the definition of the between-class scatter and the within-class scatter. The feature extraction criterion via maximizing the ratio of the between-class scatter to the within-class scatter is applied. Experimental results on the ORL, Yale, and FERET face databases demonstrate the effectiveness of the proposed algorithm.

## 1. Introduction

In the fields of computer vision and pattern recognition, many applications such as face recognition often suffer from the high-dimensional problem. In recent years, feature extraction techniques have been widely employed to reduce the dimensionality of high-dimensional data. So far, there have been a variety of feature extraction techniques. Among them, Principal Component Analysis (PCA) [1] and Linear Discriminant Analysis (LDA) [2] are two most well-known methods for linear feature extraction. PCA is an unsupervised learning algorithm, which projects the original data into a subspace spanned by the leading eigenvectors of the data's covariance matrix. Unlike PCA which is completely unsupervised with regard to the label information, LDA is a supervised algorithm, which takes full consideration of the label information. It is generally believed that label information can enhance the discriminating ability in classification tasks. Thus LDA often achieves better performance than PCA in recognition tasks. It must be noted that LDA is a linear algorithm which is designed to discover the global Euclidean structure of the data. Recent studies have shown that face image data possibly resides on a nonlinear submanifold. Thus LDA may fail to discover the intrinsic structure of the manifold. In addition, for a given test sample, the discriminant basis is whether the samples belong to the class or do not belong to another one. Each execution is based on a rigid classification principle. In fact, during the phase of feature extraction, existing training samples may be influenced by variances on facial expressions and lighting conditions, so that it is unscientific to simply sort samples into a certain class [3].

Recently, manifold learning based algorithms which are straightforward in detecting the nonlinear structures have attracted much attention of the researchers. The representative algorithms include Locally Linear Embedding (LLE) [4], Isometric Feature Mapping (ISOMAP) [5], and Laplacian Eigenmap (LE) [6]. These algorithms do yield remarkable visualization results on some benchmark data set such as facial images and handwritten digits. However, these algorithms are unsuitable for classification tasks because they are nonlinear and cannot produce effective maps for novel test data points. He et al. proposed the algorithm of Locality Preserving Projection (LPP) [7], which preserves the local structure of samples in low-dimensional feature space by projecting samples to a low-dimensional feature space with an explicit map. LPP is a linear algorithm and is able to generate effective maps for both training and test data points. Some experiments have shown that LPP can be successfully applied in face recognition. In [8], Yan et al. proposed a general framework called graph embedding for dimensionality reduction, in which all the aforementioned approaches can be reformulated. Owing to the success of LPP in face recognition, some improvements have been developed to overcome the limitations of LPP, such as Unsupervised Discriminant Projection (UDP) [9], Class-Correlation Locality Preserving Projection [10], Supervised LPP [11], Marginal Fisher Analysis (MFA) [12], Discriminant Locality Preserving Projection (DLPP) [13], Local Fisher Linear Discriminant Analysis (LFDA) [14], and Maximal Local Interclass Embedding (MLIE) [15] Many previous studies [16–18] have demonstrated that the similarity weight between samples plays an indispensable rule in describing the neighborhood relationship between samples. However, in most manifold learning algorithms such as LPP and its improved algorithms, the weight can only reflect the distance relation between samples while it neglects the practical distribution of samples.

Motivated by LDA and some other manifold learning based algorithms, this paper presents a new method called Membership-Degree Preserving Projection Analysis (MPDA) for feature extraction. MPDA utilizes the fuzzy *k*-nearest neighbor method [19, 20] to calculate the sample's membership degree with each class of samples. On this basis, the projection vector is to be figured out, minimizing the distance between the sample and the intraclass central point, as well as maximizing the distance from interclass central point. Extensive experiments on ORL, Yale, and FERET face databases show that MPDA gives better recognition result than most state-of-the-art algorithms.

The remainder of this paper is organized as follows.  Section 2 outlines LDA.  Section 3 develops the idea of MPDA and the relevant theory and algorithm.  Section 4 describes the related experiments.  Section 5 offers our conclusions.

## 2. Outline of LDA

Given a set of *n* training samples **X** = [*x*_1_,…, *x*_*n*_] ∈ ℝ^*N*×*n*^ which can be categorized into *C* pattern classes: *w*_1_ ⋯ *w*_*C*_. The basic idea of LDA is to learn a projection vector *a* to project the samples into a low-dimensional feature space such as the projected samples with maximum inter-class divergence and minimum intra-class divergence. The within-class scatter matrix *S*_*w*_ and the between-class scatter matrix *S*_*b*_ are, respectively, calculated as follows:

(1) $$\begin{array}{c} S_{w}=\frac{1}{n}\sum_{i=1}^{C}\sum_{j=1}^{n_{i}}\left(x_{j}^{i}-m_{i}\right){\left(x_{j}^{i}-m_{i}\right)}^{T}, \end{array}$$

(2) $$\begin{array}{c} S_{b}=\frac{1}{n}\sum_{i=1}^{C}n_{i}\left(m_{i}-m\right){\left(m_{i}-m\right)}^{T}, \end{array}$$

where *n*_*i*_ is the number of samples in the *i*th class, *m*_*i*_ is the mean vector of the samples in the *i*th class, *m* is the mean vector of total samples, and *x*_*j*_^*i*^ stands for the *j*th sample in the *i*th class.

LDA seeks to find a set of projection directions such that the fisher criterion (i.e., the ratio of the between-class scatter to the within-class scatter) is maximized after projection of samples. Thus the objective function of LDA can be defined by the following equation:

(3) $$\begin{array}{c} J_{\text{LDA}}=\underset{a}{\text{argmax}}\frac{a^{T}S_{b}a}{a^{T}S_{w}a}. \end{array}$$

In order to find out *d* projection vectors complying with LDA objective function, we just need to sort out the feature vectors corresponding to *d* maximum feature values of Matrix (*S*_*w*_)^−1^ *S*_*b*_.

## 3. Membership-Degree Preserving Discriminant Analysis (MPDA)

### 3.1. Basic Idea

From the literature [8], we can find that the between-class scatter matrix can be equivalently reformulated as

(4) $$\begin{array}{c} S_{b}=\frac{1}{n\left(C-1\right)}\sum_{i=1}^{C}\sum_{j=1}^{n_{i}}\;\sum_{s=1,s\neq i}^{C}\left(x_{j}^{i}-m_{s}\right){\left(x_{j}^{i}-m_{s}\right)}^{T}. \end{array}$$

According to (1), (3), and (4), it is clear to see that the essence of the fisher criterion is to minimize the distance between each sample and its intraclass center, as well as to maximize the sum distances between each sample and interclass centers. According to the previous analysis, in the procedure of feature extraction, the discriminant basis of LDA is to figure out whether the sample belongs to a certain class or does no belong to another certain class. Each execution is based on a rigid classification principle. In fact, during feature extraction, existing training samples may be influenced by different emotion and sunlight conditions, so that it is unscientific to simply sort samples into a certain class. As for this, based on LDA, the paper introduces fuzzy membership degree to describe sample distribution information. Furthermore, Membership-Degree Preserving Discriminant Analysis (MPDA) is also proposed. MPDA utilizes the fuzzy *k*-nearest neighbor method to calculate the sample's membership degree with each class. On this basis, the projection vector is to be figured out, minimizing the distance between the sample and the intraclass central point, as well as maximizing its distance to interclass central point.

### 3.2. Membership Computation

Sample membership indicates its dependence on a certain class. Defining *u*_*ij*_ as the degree that the *j*th sample belongs to class *i*, the computation of the fuzzy membership degree (*u*_*ij*_) can be realized with fuzzy *k*-nearest neighbor method [19, 20], shown as follows.

**Step 1**

Compute the Euclidean distance matrix between pairs of feature vectors in training set.

**Step 2**

Set diagonal elements of this Euclidean distance matrix to infinity.

**Step 3**

Sort the distance matrix (treat each of its columns separately) in an ascending order. Collect the corresponding class labels of the patterns located in the closest neighborhood of the pattern under consideration (as we are concerned with “*k*” neighbors, this returns a list of “*k*” integers).

**Step 4**

Compute the membership degree to class “*i*” for jth pattern using the expression proposed in the literature [20, 21]:

(5) $$\begin{array}{c} u_{ij}=\{\begin{array}{cc} 0.51+0.49\left(\frac{n_{ij}}{k}\right) & \text{if}\;\text{the}\;j\text{th}\;\text{sample}\;\text{belongs}\;\\ & \text{to}\;\text{the}\;i\text{th}\;\text{class},\\ 0.49\left(\frac{n_{ij}}{k}\right) & \text{otherwise}. \end{array} \end{array}$$

In the above expression, *n*_*ij*_ stands for the number of the neighbors of the *j*th data (pattern) that belong to the *i*th class. As usual, *u*_*ij*_ satisfies two obvious properties:

(6) $$\begin{array}{c} \sum_{i=1}^{C}u_{ij}=1,\\ 0<\sum_{j=1}^{n}u_{ij}<n. \end{array}$$

Therefore, the fuzzy membership matrix *U* can be achieved with the result of FKNN

(7) $$\begin{array}{c} U=\left[U_{ij}\right],\;i=1,2,\ldots ,c,\;j=1,2,\ldots ,N. \end{array}$$

### 3.3. The Objective Function of MPDA

In MPDA, the within-class scatter matrix and the between-class scatter matrix of samples can separately be described as

(8) $$\begin{array}{c} {\tilde{S}}_{w}=\frac{1}{n}\sum_{i=1}^{C}\sum_{j=1}^{n_{i}}u\left(i,x_{j}^{i}\right)\left(x_{j}^{i}-m_{i}\right){\left(x_{j}^{i}-m_{i}\right)}^{T},\\ {\tilde{S}}_{b}=\frac{1}{n\left(C-1\right)}\sum_{i=1}^{C}\sum_{j=1}^{n_{i}}\;\sum_{s=1,s\neq i}^{C}u\left(s,x_{j}^{i}\right)\left(x_{j}^{i}-m_{s}\right){\left(x_{j}^{i}-m_{s}\right)}^{T}, \end{array}$$

where *u*(*i*, *x*_*j*_^*i*^) stands for the membership of Sample *x*_*j*_^*i*^ to the *i*th class, while *u*(*s*, *x*_*j*_^*i*^) denotes the membership degree of sample *x*_*j*_^*i*^ to the *s*th class. The membership degrees *u*(*i*, *x*_*j*_^*i*^) and *u*(*s*, *x*_*j*_^*i*^) can be computed by using the fuzzy *k*-nearest neighbor algorithm mentioned above.

Similar to LDA, MPDA also attempts to find an optimal projection vector such that the between-class scatter is maximized and the within-class scatter is minimized after projection of samples. That is to say, the objective function of MPDA should have the following form:

(9) $$\begin{array}{c} J_{\text{MPDA}}=\underset{a}{\text{argmax}}\frac{a^{T}{\tilde{S}}_{b}a}{a^{T}{\tilde{S}}_{w}a}. \end{array}$$

In order to seek out *d* projection vectors complying with the above objective function, we just need to sort out the feature vectors corresponding to *d* maximum feature values of Matrix ${\left({\tilde{S}}_{w}\right)}^{-1}{\tilde{S}}_{b}$.

### 3.4. The Algorithm of MPDA

The algorithm of MPDA can be described as follows.

**Step 1**

For the *j*th sample *x*_*j*_^*i*^ from the *i*th class, according to fuzzy *k*-nearest neighbor method. the membership of *x*_*j*_^*i*^ with regard to the *i*th and *s*th class (*s* ≠ *i*) is figured out, respectively, denoted as *u*(*i*, *x*_*j*_^*i*^) and *u*(*s*, *x*_*j*_^*i*^).

**Step 2**

According to (8), the intraclass divergence matrix and interclass divergence matrix of sample are separately calculated.

**Step 3**

Computing the corresponding feature vectors *a*_1_ ⋯ *a*_*d*_ corresponding to the *d* maximum eigenvalues of Matrix ${\left({\tilde{S}}_{w}\right)}^{-1}{\tilde{S}}_{b}$.

**Step 4**

Sample *x* in *d*-dimension feature space is represented as *y* = *A* ^*T*^ *x*, while *A* = [*a*_1_ ⋯ *a*_*d*_].

**Step 5**

After obtaining the representation *y* of *x* in the *d*-dimensional feature space, a suitable classifer to predict the class label of *x* is adopted.

## 4. Experiment

To demonstrate the effectiveness of the proposed algorithm, MPDA, PCA, LDA, LPP, UDP, and MFA are evaluated on the ORL, Yale, and FERET face databases. After implementing the algorithms for feature extraction, the nearest neighbor classifier with Euclidean distance as a distance measure is used for classification.

### 4.1. Image Visualization

**Figure 1:**
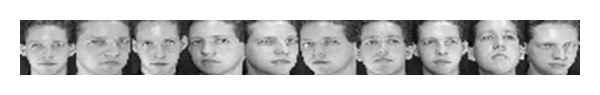
10 images of one person on the ORL database.

As human face image can be up to millions of dimensions, while human's vision is up to 3 dimensions at best, in order to more visually figure out the internal connection between the data, to compare the difference and performance of these algorithms, in the research, the four algorithms of MPDA, LPP, UDP, and MFA are selected to take image two-dimensional visualization test. The ORL database (http://www.cam-orl.co.uk) contains 400 different images from 40 subjects: each subject has 10 images. For some subjects, the images were taken at different times, with varying the lighting, facial expressions (open/closed eyes, smiling/not smiling), and facial details (glasses/no glasses). All images are grayscale and resized to a resolution of 32 × 32.  Figure 1 shows 10 images of a person on the ORL database.

**Figure 2:**
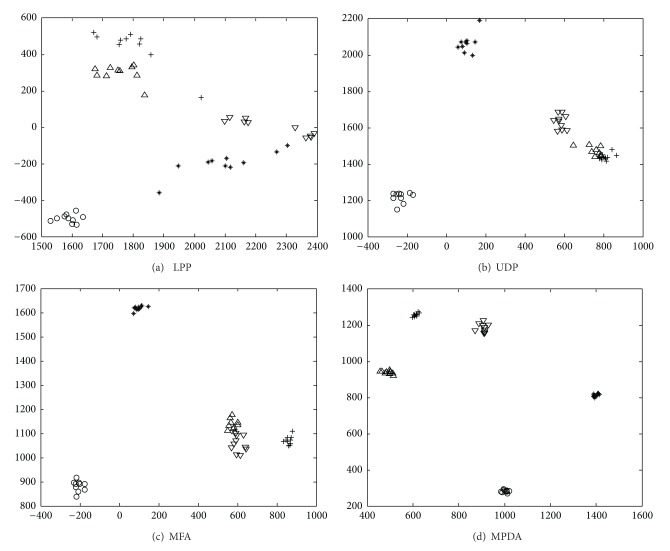
Two-dimensional projection results of LPP, UDP, MFA, and MPDA.

In this experiment, we select the images of the first five persons in the ORL database for visualization, thus the total images are 10 × 5 = 50. Let “∗,” “○,” “+,” “△,” and “▽” denote five different classes (or persons), respectively. Here, we apply LPP, UDP, MFA, and MMPDA for feature extraction, and then all the images are projected onto the 2D (two-dimensional) subspace by different algorithms.  Figure 2 shows the projection results by all the above algorithms. It can be seen from Figure 2(a) that, after projection, samples of the same class stay together, showing that LPP is able to preserve samples' local structure. Even though, LPP is not able to accurately divide samples of different classes.  Figure 2(b) shows that UDP gives consideration to samples' nonlocal structure, so that its classification performance is better than LPP. However, as UDP is a sort of unsupervised algorithm, there are still some different-class samples undivided. From Figure 2(c), we can see that MFA has taken into consideration local structure and classification information at the same time. However, it neglects sample distribution information. For this reason, there are still two classes of samples that are not clearly divided. In Figure 2(d), MPDA successfully classified 5 classes of samples, indicating that the membership degree plays an important role in feature extraction.

### 4.2. Face Recognition

**Figure 3:**
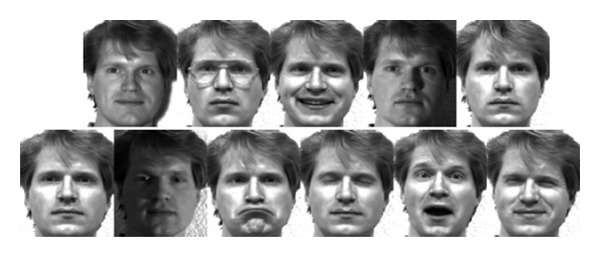
11 images of one person on the Yale database.

In order to evaluate and verify the recognition performance of MPDA compared with other methods such as PCA, LDA, LPP, UDP, and MFA, the proposed algorithm is implemented on the Yale and FERET face databases. The Yale face database (http://cvc.yale.edu/projects/yalefaces/yalefaces.html) contains 165 grayscale images of 15 individuals. There are 11 images per subject, one per different facial expression or configuration: center/left/right-light, w/wo glasses, happy, normal, sad, sleepy, surprised, and winking. All images are grayscale and normalized to a resolution of 80 × 100 pixels.  Figure 3 shows some sample images of one person from the Yale database.

**Figure 4:**
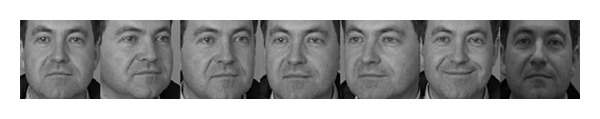
Seven images of one person in the FERET database.

The FERET database (http://en.wikipedia.org/wiki/FERET_database) contains 14126 images from 1199 individuals. In our experiments, a subset which contains 1400 images of 200 individuals (wherein each individual has seven images) is selected. The subset involves variations in facial expression, illumination, and pose. Each image is grayscale, manually cropped, and resized to 80 × 80.  Figure 4 shows some sample images of one person from the FERET database.

On the Yale database, the first *l* (=4, 5) images of each person are selected as the training set, while the rest 11-*l* images are taken as the test set. It must be noted that the PCA method is first introduced to project the original data into a subspace to overcome the small sample size (SSS) problem, where the dimension is 40.  Table 1 shows the comparison of MPDA recognition rate on two persons' faces when different parameters are configured.  Table 2 is a comparison between the best recognition rate of MPDA and other algorithms. Shown by Table 1, parameter configuration is quite critical to the recognition performance of MPDA algorithm. When *k*'s value is close to the training sample quantity of each class, MDPA has the best recognition rate. For example, when training sample quantity of each class is 4 or 5 and when *k*'s value is 5 on the Yale database, MPDA has the best effectiveness. It is shown in Table 2 that, on the Yale database, MPDA achieves the best recognition effect. The reason leading to this result lies in the following fact: compared with other algorithms, MPDA takes into consideration sample's classification information and membership information at the same time, combining sample distribution information with feature extraction, and consequently improves recognition rate.

**Figure 5:**
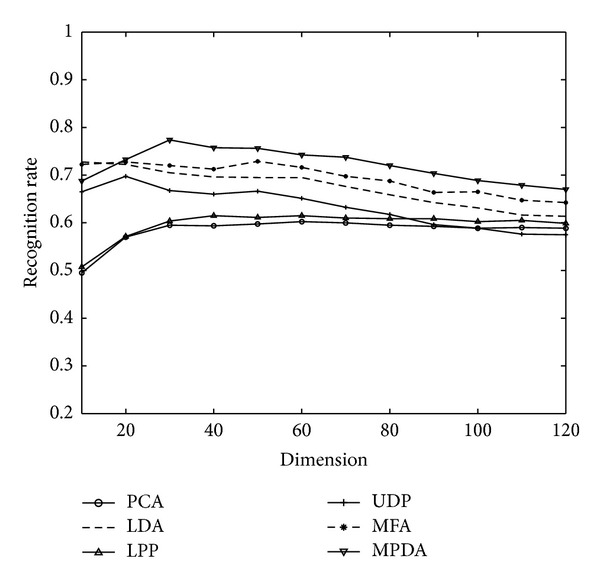
Recognition rates and corresponding dimensions of MPDA and other algorithms on the FERET database when *l* = 3.

**Figure 6:**
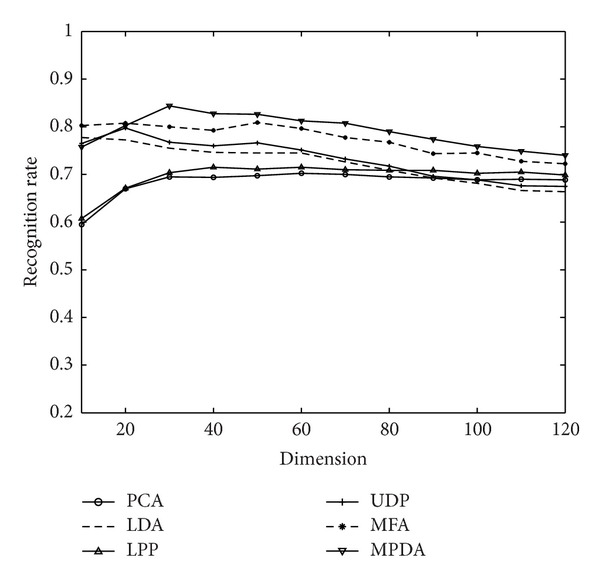
Recognition rates and corresponding dimensions of MPDA and other algorithms on the FERET database when *l* = 4.

On the FERET database, the first *l* (=3, 4) images of each person are selected as the training set, and the rest 7-*l* images are taken as the test set. Figures 5 and 6 show the comparison between the recognition rate of MPDA and other algorithms, when training sample quantity on the FERET database is separately 3 or 4 and when the projection axis of algorithm is different.  Table 3 is a comparison between the best recognition rate of MPDA and other algorithms on the three face databases. It is shown by Figures 5 and 6 that, when the projection axis quantity is higher than 30, MPDA's recognition rate is always better than other algorithms. Moreover, from Table 3, it can be seen that MPDA achieves a much higher recognition rate than other algorithms, which further demonstrates the effectiveness of the proposed algorithm, as well as the theoretical analysis based on the Yale database.

## 5. Conclusions

This paper developed a novel method MPDA for face recognition. MPDA significantly describe the internal manifold structure of the sample. In MPDA, we use the fuzzy *k*-nearest neighbor method to compute membership degree for charactering the neighboring relationship between samples and class central point. Further, we also integrate raw sample distribution information into the final feature extraction process to enhance the performance of the proposed method. Experiments on the ORL, Yale, and FERET face databases demonstrate advantages of MPDA over the other feature extraction methods.

## Figures and Tables

**Table 1:** The maximal recognition rates (%) and corresponding dimensions (shown in the parentheses) of MPDA with different *k* on the Yale database.

Training number	*k* = 1	*k* = 3	*k* = 5	*k* = 7	*k* = 9
4	91.43 (14)	95.24 (14)	97.14 (16)	96.19 (12)	96.19 (12)
5	92.22 (16)	96.67 (16)	98.67 (15)	97.33 (14)	97.33 (15)

**Table 2:** The maximal recognition rates (%) and corresponding dimensions (shown in the parentheses) of MPDA compared with other algorithms on the Yale database.

Training number	PCA	LDA	LPP	UDP	MFA	MPDA
4	91.43 (21)	96.19 (18)	95.24 (17)	96.19 (19)	96.19 (15)	**97.14 (16)**
5	92.22 (25)	95.56 (18)	96.67 (23)	96.67 (24)	97.78 (29)	**98.67 (15)**

**Table 3:** The maximal recognition rates (%) and corresponding dimensions (shown in the parentheses) of MPDA compared with other algorithms on the FERET database.

Training number	PCA	LDA	LPP	MFA	UDP	MPDA
3	60.25 (60)	72.75 (10)	61.50 (40)	72.80 (50)	69.75 (20)	**77.35 (30)**
4	70.25 (60)	77.75 (10)	71.50 (40)	80.88 (50)	79.75 (20)	**84.38 (30)**
